# Identification of Risk Factors Contributing to Prolonged Stay in the Post-anaesthesia Care Unit at a Tertiary Care Hospital in Abu Dhabi, United Arab Emirates

**DOI:** 10.7759/cureus.35741

**Published:** 2023-03-03

**Authors:** Manickam Hema Parvathy Kesarimangalam, Poornima Mahesh Hegde

**Affiliations:** 1 Department of Anaesthesia, Sheikh Khalifa Medical City, Abu Dhabi, ARE; 2 Department of Anaesthesia, Sheikh Khalifa Medical City, Abudhabi, ARE

**Keywords:** post-anesthesia discharge, surgical procedures, delay in discharge, prolonged stay, length of stay (los), post anaesthesia care unit (pacu)

## Abstract

Introduction: Post-anesthesia care units (PACU) were developed to reduce postoperative morbidity and mortality and the ideal duration of postoperative stay has been proposed as two hours; however, the incidence and risk factors for prolonged stay are variable. The objective of this study was to assess the incidence of prolonged length of stay in the post-anesthesia care unit at Sheikh Khalifa Medical City (SKMC), Abu Dhabi, United Arab Emirates, and identify the risk factors contributing to it.

Methods and Materials: This is a retrospective observational study of patients who stayed in the PACU for more than two hours. A total of 2387 patients, both male and female, who underwent surgical procedures between May 2022 to August 2022 at SKMC and were admitted to the PACU after surgery were included in the study and their data were analyzed.

Results: Of the 2387 patients who underwent surgical procedures, 43 (1.8%) had prolonged stays in the PACU. Of these, 20 (47%) were adult cases and 23 (53%) were pediatric cases. The main reasons for the delay in discharge from PACU in our study were the non-availability of ward beds (25.5%), followed by pain management (18.6%).

Conclusions: We recommend improving the communication between different specialties, restructuring staffing, implementing changes in perioperative management, and changing operating room scheduling to prevent avoidable reasons contributing to a prolonged stay in the PACU.

## Introduction

Post-anaesthesia care units (PACUs) were established in 1923, with the primary aim of reducing postoperative morbidity and mortality. Standardizing the appropriate and average length of time of PACU stay is difficult, as there is no literature that describes an ideal length of stay based on the recovery of the patient. [1]. The actual PACU length of stay is determined from the time the patient was admitted to the PACU postoperatively to the time they are medically stable for discharge from the PACU, as recorded by the PACU nurse. PACU length of stay is considered as a clinical indicator [2] and various studies have shown that patients achieve satisfactory discharge scores within two hours of the postoperative period [1]. 

The objective of this study was to evaluate the incidence of and the factors responsible for a prolonged stay in the PACU.

## Materials and methods

This is a retrospective audit, and the data reviewed in this article were collected over a period of four months from May 1, 2022, to August 1, 2022, at Sheikh Khalifa Medical City Hospital (SKMC), Abu Dhabi, United Arab Emirates; the data were retrieved from the hospital records. Ethical approval for this study was provided by the Institutional Review Board/Research Ethics Committee of SKMC on October 26, 2022 (approval number: REC-25.10.2022[RS-772]).

In our institute, we use the modified Aldrete score for evaluation and discharge of the patient from the PACU unit, where the vitals are recorded every 10 minutes and in case of suspicious airway problems, it is recorded every five minutes. Patients should have a score of 9/10 or above to be discharged from the PACU unless approved by the anaesthetist.

All adult and paediatric patients who stayed in the PACU for more than two hours postoperatively in the PACU were included in the study. A total of 2387 patients underwent surgical procedures at SKMC during the study period, out of which 43 patients had a prolonged PACU stay. The details of these patients were analysed to identify the factors contributing to prolonged PACU stay. 

The data collected included preoperative variables such as age, gender, American Society of Anaesthesiology (ASA) status, comorbidities, intraoperative variables such as type of anaesthesia and operative procedure, and postoperative variables such as cardiovascular, respiratory, and neurological adverse effects. Also included were variables such as pain management, postoperative nausea and vomiting (PONV), unplanned admission, waiting for primary physician (surgeon) orders, and bed availability. These were compared with the studies conducted in other hospitals. 

## Results

**Figure 1:**
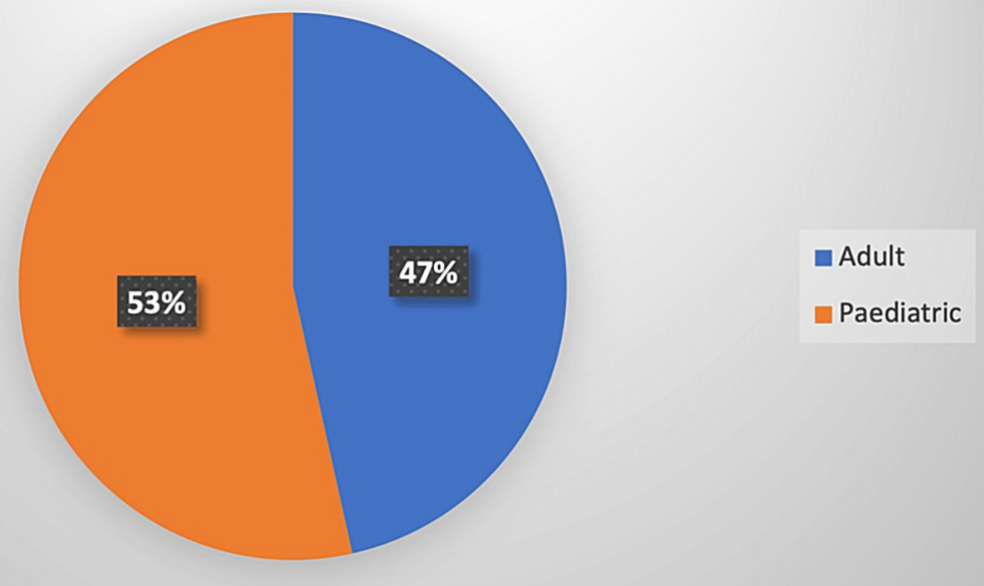
Prolonged stay in PACU in adult and pediatric cases PACU: post-anaesthesia care unit

A total of 43 patients had prolonged PACU stay in our hospital. Of these, adult cases were 20 (47%) and pediatric cases were 23 (53%) as shown in Figure 1.

**Figure 2:**
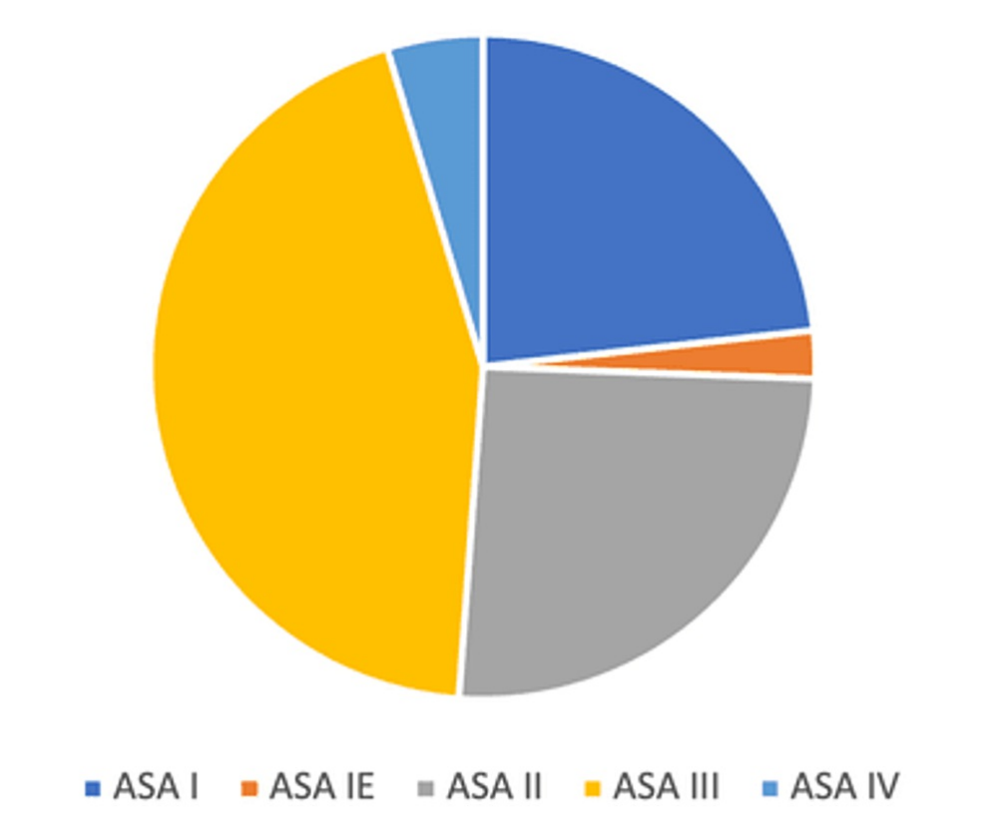
ASA scores of operated patients ASA: American society of Anaesthesiology

The ASA scores of the 43 patients with prolonged stay in PACU were as follows: 10 (23%) patients of ASA I, one (2%) patient of ASA IE, 11 (26%) patients of ASA II, 19 (44%) patients of ASA III, two (5%) patients of ASA IV. The distribution of ASA scores of operated patients is shown in Figure 2.

The speciality-wise breakdown was as follows: urology (20.9%), ENT (18%), orthopaedics (16.2%), general surgery (13.9%), maxillofacial (11.6%), and paediatric surgery (11.6%), followed by (2.32%) each by dental, gynaecology, and pulmonology (Table 1). Among the nine urology cases, three patients (14.3%) were in the PACU for pain management and two patients (9.5%) were waiting for surgeons' postoperative orders. The other reasons for prolonged stay among urology patients were observation for a longer time for postoperative bleeding, for control of blood pressure, waiting for transport to be shifted to another facility, and waiting for the availability of ward beds.

**Table 1:** Breakdown of cases of prolonged stay in the PACU according to specialty PACU: post-anaesthesia care unit

Speciality	Number of patients
Urology	9
ENT	8
Orthopaedic surgery	7
General surgery	6
Maxillofacial surgery	6
Pediatric surgery	5
Dental surgery	1
Gynaecology	1
Pulmonology	1

The main factors identified for a prolonged stay in the PACU were unavailability of ward bed (11 patients), requiring pain management (eight patients), haemodynamic instability (four patients), desaturation (four patients), delay in waking up (three patients), observation (three patients), waiting for an ambulance (three patients), elevated blood pressure (three patients), waiting for surgeon’s order (two patients), and postoperative bleeding (two patients). The data is given in Table 2.

**Table 2:** Reasons for prolonged stay in the PACU summarized in the order of decreasing incidence PACU: post-anaesthesia care unit

Reason for prolonged stay	Patients, n (%)
Unavailability of bed in the ward	11 (25.5%)
Pain management	8 (18.6%)
Haemodynamically unstable	3 (9.3%)
Desaturation	4 (9.3%)
Delay in waking up	3 (6.97%)
Waiting for ambulance	3 (6.97%)
Observation	3 (6.97%)
Elevated blood pressure	3 (6.97%)
Waiting for surgeon’s order	2 (4.65%)
Postoperative bleeding	2 (4.65%)

In our study of the patients in the ASA III group, the majority were paediatric patients (68.42%). Of these, 73.6% stayed for a longer duration for medical reasons, and 26.3% stayed longer waiting for ward beds. Of the patients waiting for ward beds, 10.5% of patients were requested by primary surgeons for close observation. The majority of the paediatric ASA III patients were posted for ENT surgery followed by orthopaedic limb correction surgery mainly in children with special needs.

Of the two ASA IV patients, one had a motor vehicular accident posted for open reduction and internal fixation (ORIF) mandible with very small pneumothorax, without fracture of the ribs. Postoperatively, he developed tachypnoea and tachycardia, and the postoperative radiograph was suggestive of increasing pneumothorax, so was shifted to the intensive care unit. The other patient was in PACU for a post-caesarean section for an exit procedure (foetal neck hygroma) and developed postoperative bleeding requiring close monitoring and blood transfusion.

## Discussion

The main purpose of this study was to identify the factors responsible for delay in PACU discharge and use these data to identify the clinically relevant factors for evaluating the efficacy of care. Weissman et al. stressed that delay in discharge from PACU results in congestion and bottleneck at various stages of perioperative care and increases the workload of nurses [3]. Barash et al. [4] and Collin et al. [5] also stress that delay results in an increase in expenses significantly and length of stay is an important outcome as a marker for resource consumption.

In our study, the incidence of prolonged PACU stay was 1.8%, which is much lower than that reported by Samad et al. (8.1%) and by Anwar et al. (2.5%) [6,7]. Most patients included in the study belonged to ASA III (44%), followed by ASA II (26%), ASA I (23%), ASA IV (5%) and ASA IE (2%). These data suggest that a greater number of patients belonged to ASA III, which corresponds with the findings from other large-group prospective studies [8-10]. This shows that preoperative factors such as age >60, ASA III, having comorbidities such as obesity, hypertension, diabetes, intraoperative blood transfusion, and duration of surgery greater than three hours showed prolonged PACU stay. It is well known that airway procedures, especially procedures like adenoidectomy and tonsillectomy in paediatric patients with comorbidities increase the chance of prolonged PACU stay [11]. 

Our study suggests the main reason for the delay in PACU discharge is the unavailability of ward beds (25.5%), This result is comparable to a prospective study conducted by Samad et al., which showed unavailability of beds as the main reason for the delay in discharge from PACU [6]. Another prospective study by Cowie et al. also showed that delay in discharge was due to ward bed unavailability (52%) [12], which was much higher than in our study group. The unavailability of the ward beds was because it was not booked preoperatively, unplanned admission, or hospital beds were full. In the current study, out of the 11 patients waiting for ward beds, only three patients had unplanned admission to the ward for medical reasons (9%) or social reasons (on request by the parents) (18.1%).

The second most common reason for the delay in transfer from PACU was pain management (18.6%), which is comparatively higher than that reported in other prospective studies [6]. A large clinical postoperative pain trial by Chan et al [13] and another prospective study by Aubrun et al. [14] suggested that younger patients, females, abdominal surgeries, and orthopaedic surgeries have more severe pain in PACU, which can prolong the stay. In our study, the postoperative pain was similar in urology (37.5%) and orthopaedics (37.5%) patients. Postoperative pain was the reason for prolonged PACU stay for one patient (12.5%) who underwent surgery for a cleft palate and one patient (12.5%) who underwent laparoscopic appendectomy. 

All orthopaedic patients who required prolonged stay in the PACU due to postoperative pain were paediatric patients for lower limb reconstruction surgery. Of the three orthopaedic patients, only one had general anaesthesia (GA) with regional anaesthesia, while the other two patients had GA without regional block. In urology, all three patients were adults, two of whom had laparoscopic nephrectomy (donors) and one patient had Baori flap reimplantation. The incidence of postoperative pain can be reduced by regional techniques, pre-emptive analgesia, and multimodal analgesia. At the time of this study, we, in our hospital, had started performing most of the paediatric orthopaedic cases under GA with blocks. 

The cardiovascular and respiratory event rate in our study was 25.5%, while another retrospective large group study by Anwar et al. [7] showed that the postoperative cardiovascular and respiratory event rate accounting for delay in discharge from PACU was 40.33%. The cardiovascular and respiratory events were comparatively fewer in our study. Adverse respiratory effects occurred in our study mainly in the paediatric age group, with comorbidities posted for airway procedures. Cardiovascular events in our study occurred more frequently in adult patients with a history of hypertension and diabetes, and two patients with obesity.

In a large retrospective study by Weingarten et al., PONV was the most common factor for prolonged discharge from the PACU [15]. None of the patients in our study had PONV as a cause of delayed discharge from the PACU.

A limitation of this study is that the data collected were small and over a short duration. Larger studies over a longer period would provide more insight into the incidence and analysis of preventable causes of prolonged PACU stay.

## Conclusions

Prolonged stay in PACU has an impact on both quality of care and management of flow of patients in operating rooms. The main reasons for prolonged stay in our study were waiting for ward bed availability, followed by pain management. We recommend improving the communication between different specialities, restructuring staffing, implementing changes in perioperative management, changing operating room scheduling and lowering the incidence further by initiating measures to prevent avoidable reasons contributing to prolonged stays.
